# A silk fibroin based green nano-filter for air filtration†

**DOI:** 10.1039/c7ra12879g

**Published:** 2018-02-20

**Authors:** Xiaochao Gao, Jing Gou, Ling Zhang, Shasha Duan, Chunzhong Li

**Affiliations:** School of Materials Science and Engineering, Key Laboratory for Ultrafine Materials of Ministry of Education, East China University of Science & Technology 130 Meilong Road Shanghai 200237 China czli@ecust.edu.cn

## Abstract

Fibrous air filters fabricated by electrospinning have proved to be an effective approach among the various strategies for PM2.5 removal. However, in the electrospinning process, the large amounts of toxic organic solvents usually evaporate into the atmosphere and disposing of these used polymer-based air filters would leave further pollution in the environment. Here, we report on the fabrication of a silk fibroin based nanofiber air filter with robust filtration performance *via* a green electrospinning process. Silk worm cocoons were degummed and dialyzed against water to form the silk fibroin solution and then the silk fibroin nanofiber membranes were fabricated by electrospinning with the help of polyethylene oxide. Moreover, special attention was paid to the morphological evolution of the pollutants captured by the nanofiber nets during the filtration process. It was discovered that the inherent properties of silk fibroin play a key role in improving the filtration performance. Benefiting from the richness of functional groups, the resultant silk fibroin fibrous membranes exhibited a high filtration efficiency of 99.99% with a relatively low air resistance of only 75 Pa, leading to an obvious higher quality factor. Due to the biodegradability of silk fibroin, the membranes are disposable after use. We believe that the methodology and results presented here will not only provide a novel perspective for air filtration, but also pave the way for producing a safe and clean air filtration system.

## Abstract 2

This paper reports the fabrication of a silk fibroin nanofiber air filter *via* a green electrospinning method.

## Graphical abstract

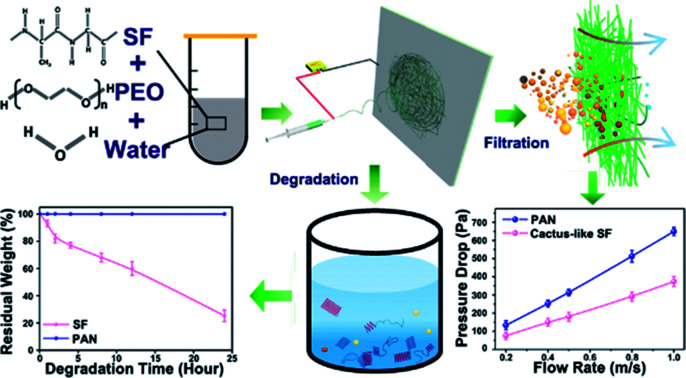

## Introduction

**Fig. 1:**
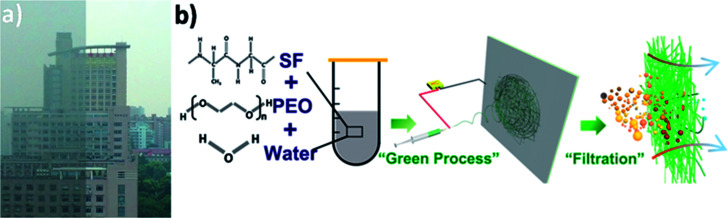
(a) Photograph of an identical place during clear and hazy days in Shanghai and (b) schematic of fabricating silk fibroin nanofiber membranes for air filtration.

Air pollution is growing worse in certain parts of the world, especially in Asia, Africa and Latin America, hitting the developing countries hardest and resulting in a wide range of potentially life-shortening health problems. Now, the devastating air pollution poses a great threat to human beings, climates and ecosystems and this trend is expected to continue in the future.^1–7^ A photo taken of the same place during clear and hazy days indicates that visibility decreases greatly due to air pollution (Fig. 1a).

Airborne particulate matter (PM), a major type of anthropogenic air pollutant, is a complex mixture of inorganic matters (including sulfur dioxide, oxides of carbon, oxides of nitrogen) and organic matters (including volatile organic compounds and elemental carbon).^1^ Based on its aerodynamic diameter, PM can be classified into fine particulate having an aerodynamic diameter < 2.5 μm (PM2.5) and coarse particulate having an aerodynamic diameter < 10 μm (PM10). They are both composed of a variety of components, but differ vastly in physical properties, health risk and behavior in the atmosphere. PM2.5 is of particular concern as some components of it can penetrate deeply into lung alveoli and enter into bloodstream.^8^ Moreover, PM2.5 has the longest atmosphere lifetime (days to weeks), and thus can accumulate and spread by air flow to extended area. Extensive studies have reported that cardiovascular problems can be induced within hours of inhalation of PM2.5, and chronic exposure leads to a reduction of several years in life expectancy. PM is believed to be the most deleterious air pollution in affecting human health and it has been proved to be responsible for 3–7 million deaths worldwide per year.^9–13^ PM is mainly generated in the process of urbanization and industrialization, including incomplete combustion of fossil fuel, biomass burning, and vehicle emissions.^14,15^

In current situation, many methods have been developed and applied to relieve people from air pollution. Individuals can take a commercial breathing mask to prevent the inhalation of PM2.5 when taking outdoor activities and in indoor spaces, air quality is ensured by employing the electrostatic air cleaners or modern ventilation systems. However, the function part of conventional air filter is mostly made of several layers of randomly oriented melt blown micron-scale fibers, which is inherently incapable of capturing fine particles due to the filtration mechanism.^16^ Fortunately, if the diameter of the filtration media decreases to some degree, the formed sinuous channels will become narrow enough to trap PM2.5 particles, let alone the PM10 particles. Therefore, benefiting from the relatively small diameter, nanofibers are attracting great attention in the field of air filtration.^16^ Electrospinning, as a promising technology to fabricate nanometer-scale fibers in large scale efficiently, has stand out compared with various routes such as template synthesis,^17^ phase-separation,^18^ hydro-thermal reaction,^19^*etc.* Based on the electrospinning technology, a variety of polymeric nanofibers have been prepared for air filtration, including polyurethane,^20^ polyacrylonitrile,^21^ polysulfone,^22^ poly(lactic acid),^23^ polycarbonate,^24^ poly(vinyl alcohol),^25^ polyamide,^26^*etc.* However, to make these synthetic polymers electrospinnable, large amount of organic solvents is used to dissolve them. The decent concentration of an electrospinning solution is usually less than 30%, indicating that most of the toxic, harmful organic solvents will evaporate into the atmosphere. Besides, once the air filter reaches the point where they can no longer be used, it will be disposed as non-recycled plastic waste, and then burned in waste-to-energy power plants or ends up in landfills where it may take up to 1000 years to decompose. Considering the source of PM pollution and the two aforementioned points, the demand for advanced filtration media research that looks toward the use of green technologies and alternative to synthetic materials increases.

Silk is a semi-crystalline biopolymer produced by silkworms. Silk fibroin (SF), a natural protein extracted from the *Bombyx mori* silk worm cocoon,^27^ has been used for clothing in ancient China and been researched in various fields in recent years due to its several distinctive properties including mechanically robust strength,^28^ good biocompatibility, lack of toxicity^29^ and aqueous processibility.^30^ Up to now, only a few investigation reports the use of silk in the field of air filtration. Zhang *et al.*^31^ demonstrated a human-friendly silk nanofiber air filter fabricated by electrospinning from SF/formic acid precursor solution. T. Scheibel *et al.*^32^ produced silk-based fine dust filters for air filtration by using hexafluoroiso-2-propanol (HFIP) as solvent, which is more toxic and dangerous. Clearly, the above studies have several drawbacks: (i) the inescapable use of harmful organic solvents that determined by the electrospinning mechanism, (ii) not elucidating the mechanism resulting in the difference between synthetic polymers and SF in filtration performance.

Here, we present the fabrication of SF-based fibrous filtration media with robust filtration performance *via* green electrospinning process (Fig. 1b), which is a significant step toward safe and clean fabrication. To avoid the use of toxic and harmful organic solvents, the SF-based nanofiber membranes were fabricated by electrospinning from a non-toxic precursor solution (mixture of water, silk fibroin, and PEO). More significantly, the effect of fiber surface structure and property on the time evolution of filter clogging were investigated. Lastly, the biodegradability of SF-based membranes was studied. It has been found that SF-based nanofiber membranes possess a high filtration efficiency of more than 99.99% and a low pressure drop of 75 Pa. As an approach that is cost-effective, environment-friendly and scalable, the SF-based fibrous filtration media not only provides a novel perspective for air filtration, but also paves the way for producing a safe and clean air filtration system. The main text of the article should appear here with headings as appropriate.

## Experimental

### Materials

Silk cocoons were purchased from Huzhou, Zhejiang province, China. Polyethylene oxide (PEO), polyacrylonitrile (PAN) and lithium bromide (LiBr) were purchased from Sigma-Aldrich. *N*,*N*-dimethylformamide (DMF) were obtained from Shanghai Chemical Reagents Co., Ltd, China. All chemicals were of analytical grade and used without further purification.

### Preparation of silk fibroin solution

Silk fibroin was extracted from *Bombyx mori* silk worm cocoons according to previously published methods.^30^ Cocoons were removed of the insect and immersed into boiling Na_2_CO_3_ solution (0.02 M) for 60 min. The degummed fibers were rinsed with distilled water to remove residual Na_2_CO_3_ solution and air dried overnight. The dried fibers were solubilized in LiBr (9.3 M) at 60 °C for 4 h. The obtained solution was dialyzed against distilled water with a regenerated cellulose tube (3500 g mol^−1^ molecular weight cut off). The water was changed every 6 hours for 12 times. The solubilized silk fibroin protein solution was dialyzed against pure water for 36 h to remove LiBr, and then centrifuged to remove insoluble particulates and stored at 4 °C. The final silk fibroin concentration was about 8% (w/v) after purification.

### Preparation of electrospinning precursor solutions

The 8 wt% silk fibroin electrospinning precursor solution was prepared by adding PEO (1 000 000 g mol^−1^) directly into the silk aqueous solutions to generate SF/PEO solutions with the weight ratio of 1 : 4. PAN was dissolved in DMF to a concentration of 10 wt% for electrospinning.

### Preparation of fibrous membranes

Different kinds of electrospinning precursor solutions were loaded in a 5 ml syringe, and a positive DC voltage (10–20 kV) was applied at the stainless steel needle tip and collector (connecting earth). The syringe pump pushed the solution through the needle tip slowly. The applied potential, feeding rate, electrospinning duration and needle-collector distance were carefully adjusted according to different kinds of precursor solutions. Lastly, SF/PEO, PAN fibrous membranes were collected on the aluminum foil collectors after 10–50 min of electrospinning. For SF/PEO nonwoven fabrics, they were immersed into an ethanol solution for 10 min at room temperature and then incubated in water for 36 h at 37 °C to remove PEO.

### Characterization

The morphological features of SF/PEO, PAN, and commercial fibrous membranes were characterized by Hitachi S-4800 cold-cathode field-emission scanning electron microscope (FE-SEM). Fourier transform infrared spectroscopy (FTIR) (Nicolet 5700) was conducted in the transmittance mode in the spectral range of 800–4000 cm^−1^ with a resolution of 0.1 cm^−1^. Water contact angle test was carried out using HARKE-SPCAX3 contact angle measuring instrument.

### Filtration test

The samples were detached from the aluminium foils and then attached on the hole of the supporter to block the diffusion of smoke. The PM2.5 was generated by burning of cigarette and collected in the reactor under the presence of moisture. A particle counter (Lighthouse 3016) was used to measure the PM particle number concentration with and without filters. The pressure drop was measured by a differential pressure gauge (Summit digital Manometer 635).

## Results and discussion

**Fig. 2:**
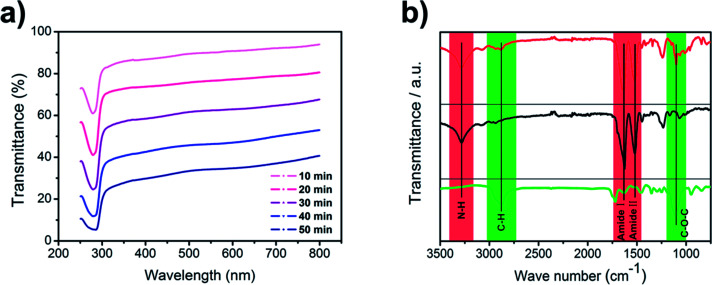
(a) Transmittance spectrum of the silk fibroin nanofiber membranes. (b) FT-IR spectrum of SF/PEO blend nanofiber (upper red line), PEO extracted SF nanofiber (medium black line), and pure PEO nanofiber (lower green line).

Electrospinning has been intensively studied to prepare nanofibers from different polymer solutions. We added PEO into silk fibroin solution to improve the electrospinnability because viscosity plays an important role in the electrospinning process (Fig. 1b). The viscosity of pure silk fibroin solution is relatively low that the balance between the viscoelastic force, surface tension and electrostatic repulsion is easy to destroy, so the jet would break into droplets instead of forming a continuous fiber. The feeding rate, applied voltage at the tip and tip-to-collector distance were finely adjusted to generate uniform nanofibers. The transmittance curves with the electrospinning time ranging from 10 min to 50 min are shown in Fig. 2a. The nanofiber membranes have a transmittance over 50% at most visible region when the corresponding electrospinning time is less than 30 min. After the nanofiber membranes were collected on the nylon fiber nonwoven frame (possesses negligible filtration efficiency and pressure drop), they were immersed in an ethanol solution for 10 min and then incubated in distilled water for 36 h at room temperature to remove PEO. The ethanol annealing process was carried to induce the transition of β-sheets, preventing the silk fibroin phase from dissolving in water and contributing to the superior strength of silk fibroin nanofiber.^33^ After the treatment with water, the membranes were dried in a vacuum at room temperature.

FTIR spectra of SF/PEO blend nanofiber, PEO extracted SF nanofiber, and pure PEO nanofiber are shown in Fig. 2b. For pure PEO, the main peaks are at 2874 cm^−1^ and 1104 cm^−1^ due to C–H stretching and C–O–C stretching vibrations. For samples containing SF, the characteristic β-sheet peak was observed at 1516 cm^−1^ (Amide I) and 1628 cm^−1^ (Amide II) while the N–H stretching vibration was observed at 3282 cm^−1^ respectively, indicating the existence of amide bond. The FTIR spectra of PEO extracted SF did not show the feature peaks at 2874 cm^−1^ and 1104 cm^−1^, confirming that PEO are extracted from the SF/PEO blend fiber after incubating in water and the peaks at 1516 cm^−1^ and 1628 cm^−1^ indicated that SF was reserved. These results demonstrated that after incubating in water for 36 h, PEO phase was successfully removed from the SF/PEO blend nanofiber.

**Fig. 3:**
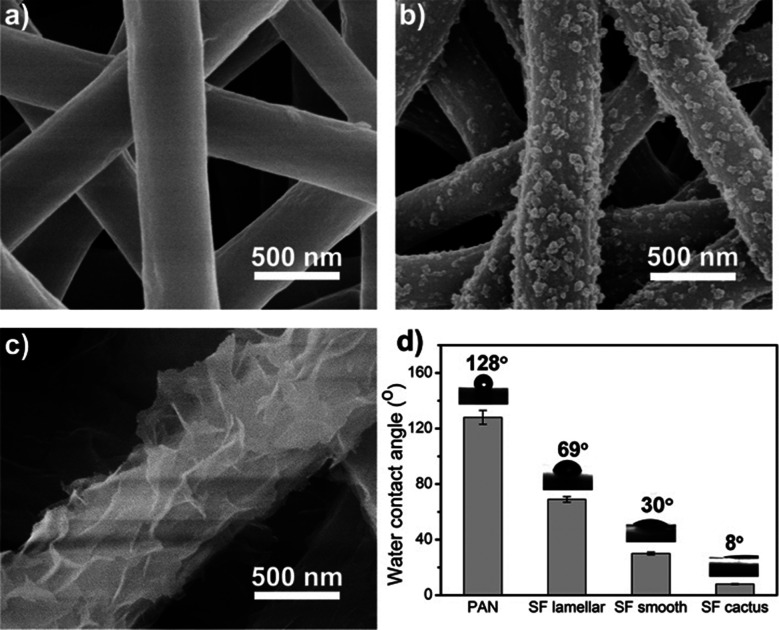
FE-SEM images of SF/PEO blend nanofiber after incubating in water for 0 h (a), 36 h (b) and 60 h (c). (d) Water contact angle of different kinds of nanofiber membranes.

Besides the FTIR analysis, morphological changes of SF/PEO blend nanofiber after incubating in water were further examined by FE-SEM (Fig. 3a and b). The diameter of SF/PEO blend nanofiber produced from SF solution was about 400 nm in average. When the SF/PEO blend jet was stretched from the Taylor cone, a thin skin layer formed at the liquid–gas interface, which is enriched with PEO.^34^ After water incubation, the PEO phase first dissolved then the surface of fiber became rough and the diameter contracted a little (Fig. S1, ESI†). The size of these randomly spread globules on the surface were about 20–80 nm. The cactus-like structure provides the fiber with higher surface energy and improve the wettability of the nanofiber membrane, which will be confirmed in the water contact angle test. When the water incubation time increases to 60 h that permits sufficient time for the hydrophilic fibroin chain block dissolve, the remaining crystalline part will self-assembly into a lamellar-like structure (Fig. 3c), finally transforming into a hydrophobic surface.

To compare the morphology, surface property and filtration performance of various fibers, the mostly studied PAN nanofibers were fabricated *via* electrospinning as well. By changing the applied voltage, electrospinning time, solution concentration, and the distance between the tip and collector, the diameter and pore size distribution of PAN nanofibers were finely tuned according to the SF-based one. The diameter of the electrospinning fiber increased with a higher feeding rate or higher concentration. More details could be found in the Methods part. PAN nanofibers membranes were fabricated by a traditional electrospinning process using dimethylformamide as solvents and then transported to a nylon nonwoven frame to form the air filter.

For all these fibrous air filters with a same diameter and pore size distribution, the wettability was examined by water contact angle measurement. Water contact angle indicates the wettability of a membrane surface, and an ideal filtration membrane is one that produce a high flux without clogging or fouling. The water contact angle of different kinds of nanofiber membranes were summarized in Fig. 3d. The pristine PAN fibrous membrane showed a hydrophobic surface whereas all silk fibroin membranes showed a good hydrophilicity. Fig. 3d clearly shows that the water contact angle of PEO extracted silk fibroin nanofiber membranes is almost constant up to 8 degrees, which indicates excellent hydrophilicity. The effect of surface wettability on the filtration performance will be discussed in the next section.

### Filtration performance

To better understand this section, the reader is recommended to read this article,^35^ which has thoroughly characterized the physical and chemical properties of the most universal and representative PM pollutants. In this study, the PM was generated by burning cigarette since the ingredient of smoke generated by the cigarette is similar to that of PM pollution in the air.^36^ The particle size distribution of the smoke generated by burning cigarette was measured by a particle counter, which shows a broad distribution from 0.3 μm to 10 μm and a large proportion of PM2.5 (Fig. S3b, ESI†). The concentration of the smoke in the left cabin was diluted with clean air to a severe level, where the air quality index (AQI) reading was at 500. The nanofiber membranes were pasted between the cabins, blocking the diffusion of smoke from the left cabin to the right one (Fig. S3c, ESI†). We also cut a piece of commercial air filter to evaluate the filtration performance.

**Fig. 4:**
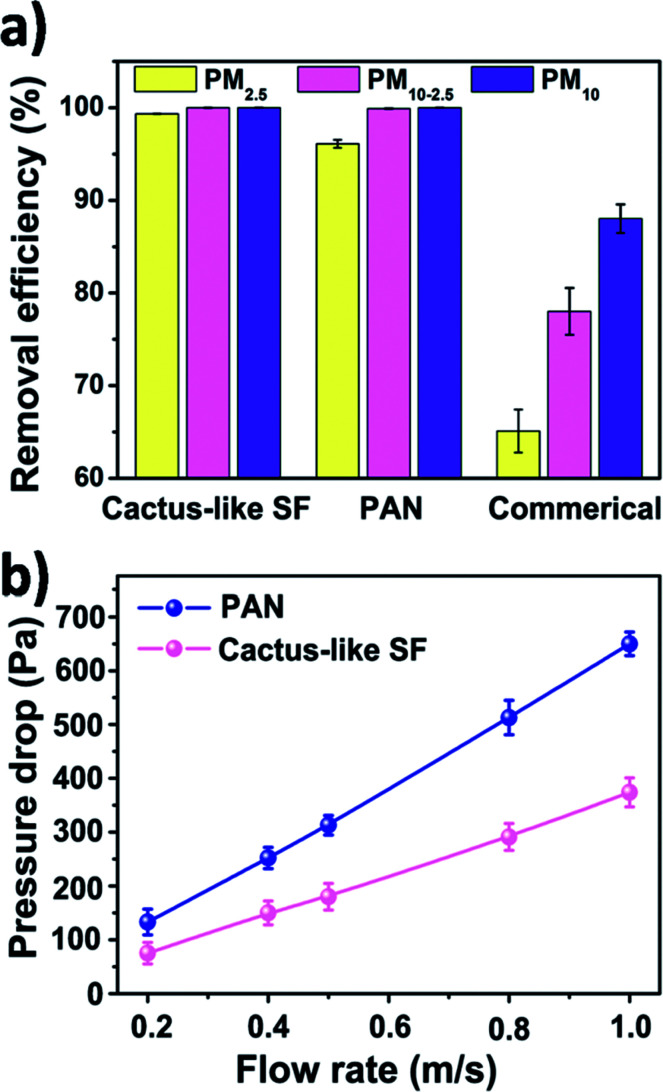
(a) Filtration efficiency of cactus-like silk fibroin, PAN, and commercial air filter. (b) Pressure drop of cactus-like silk fibroin and PAN nanofiber air filter under different air flow rate.

The filtration results were shown in Fig. 4a. All air filters share a similar thickness and pore size distribution (pore distribution of SF and PAN nanofiber membranes are shown in Fig. S2, ESI†). The removal efficiencies are calculated by measuring the PM particle number concentration with and without air filters. It could be clearly seen that for PM10-2.5, silk fibroin and PAN nanofiber air filter exhibited a good removal efficiency of 99.99% and 99.89%, respectively. While the removal efficiency of the commercial filter was only 88.02% for PM10-2.5. Data in the PM10-2.5 and PM10 columns indicates that no matter what material the nanometer-scale fibrous filters is made from, they all showed a higher removal efficiency (>99%) for coarse particles than the commercial air filter. This is because of the size-based filtration mechanism of the fibrous filtration system.^16^ Not taking the gravity effect into account, there are four main filtration mechanisms of fiber material: interception, Brownian diffusion, inertial effect and electrostatic effect. For coarse airborne pollutants, they will be trapped easily by the interception and inertial impaction from the nanofiber air filter. However, they can still penetrate the sinuous channels formed by micron-scale fiber membrane. The surface morphologies of different filters before and after filtration were studied by using a FE-SEM (Fig. S3c, ESI†). After the filtration, PM particles were obstructed by the nanofiber net and the fiber diameter increased. However, the commercial air filter showed a much larger fiber diameter and the capture of PM was much less than those we fabricated by electrospinning. For particles having an aerodynamic diameter < 2.5 μm, which does not have enough inertia to be captured by interception impaction and is mainly captured by the interaction between fiber and particles, the surface property of different filters counts. It is noted that silk fibroin nanofiber air filter showed an enhanced filtration efficiency for PM2.5, increasing from 96.11% to 99.84% compared with the synthetic polymer filter.

In addition to filtration efficiency, pressure drop is another important parameter for air filters, as the pressure drop is related to the maintenance and remaining use time of the filter. Here, we investigated the pressure drop of silk fibroin nanofiber filter and the PAN one under different air flow velocities. Fig. 4b shows the relationship between the pressure drop and the velocity of the air flow. As the filter face velocity increased from 0.2 to 1 m s^−1^, the pressure drop of cactus-like SF nanofiber filter increased linearly from 75 to 374 Pa and the pressure drop of PAN one increased from 133 to 650 Pa, respectively. The pressure drop of SF-based membranes is only half of that of PAN. The result is consistent with the theoretical equation that the pressure drop increases with increasing the air flow velocity:1
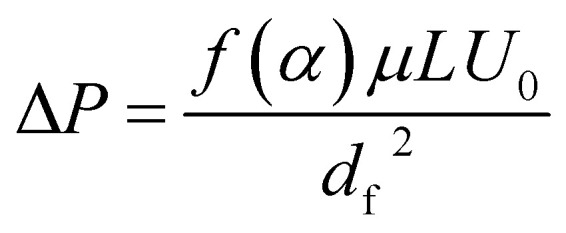
where *μ* and *U*_0_ are gas viscosity and velocity, *L* and *d*_f_ are the thickness of the filter and diameter of fiber, respectively, and *f*(*α*) represents the pore size distribution.^37^ Interestingly, the silk fibroin nanofiber filters possessed a relatively lower pressure drop than the PAN one although they shared a similar fiber size and packing density. This implied that there were other undetected but critical factors that control the air flow resistance.

To quantitatively compare the filtration performance of the SF-based nanofiber membrane and PAN nanofiber membrane, the quality factor was calculated after each experiment. The quality factor (QF) is a standardized parameter that correlates the removal efficiency and pressure drop to evaluate the filtration performance of an air filter, QF = −ln(1 − *E*)/Δ*P*, where *E* is removal efficiency and Δ*P* is the pressure drop. As the air flow velocity increases, the QF of SF-based nanofiber membrane increases more steeply than that of PAN nanofiber membrane (Fig. S3d, ESI†). The above results indicate that the SF-based nanofiber membrane is able to improve the filtration efficiency of PM pollutants while decreasing the air-flow resistance.

**Fig. 5:**
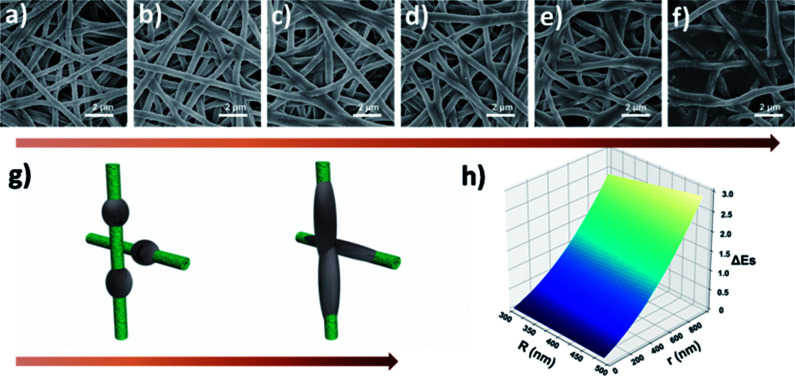
(a–f) SEM images of fluffy soots captured by silk fibroin nanofiber membranes under a continuous flow of smoke at different time sequences. (g) Schematic illustration of the capture, deformation, coalescence, and growth of the fluffy soots on silk fibroin nanofiber nets. (h) 3D surface plot of the energy change during the filtration process.

To further understand the filtration performance improvement of SF-based fibrous filtration media, special attention was paid to the morphological changes of the fluffy soot aggregates during the filtration process. Concretely, FE-SEM was employed to observe the morphologies of PM captured by nanofiber membrane at different time during a continuous filtration process. Fig. 5a shows the SF-based nanofiber membrane before capturing PM pollutants and the time sequence of PM capture is shown in panels (a–f) in Fig. 5. At the initial stage, as the smoke flowed past the nanofiber net, PM particles were randomly captured by the fiber and wrapped tightly on the nanofiber net. Subsequently, as the capture kept going on, the particles captured on the nanofibers are able to deform and spread over the nanofiber surface, without forming any clogging and destroying the tortuous channel. Finally, the cactus-like structure became smooth and the diameter increased uniformly from 400 nm to 600 nm after capturing the PM particles. These observations demonstrate that the PM pollutants could deform and spread on the silk fibroin nanofibers. Schematic illustration of the capture, deformation, coalescence, and growth of the fluffy soot aggregates during the filtration process was shown in Fig. 5g. The shape variations were mainly determined by the competition between the surface tension of pollutants and the adhesion between pollutants and nanofibers. For the pollutants captured by the filtration membranes, the general form of the energy is2
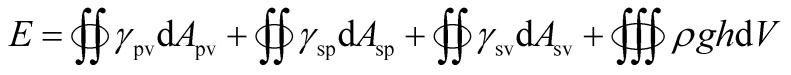
where *γ*_pv_, *γ*_sp_, and *γ*_sv_ are the interfacial tension of pollutant-vapor, solid-pollutant, and solid–vapor, respectively. *A*_pv_, *A*_sp_, and *A*_sv_ refer to the contact area between the pollutant-vapor, the solid-pollutant, and the solid–vapor interfaces. The fourth term represents the gravitational potential energy and once the pollutants are captured by the nanofiber, the variation of gravitational potential energy can be neglected. According to the principle of energy minimization, the surface energy released in the deformation process is

3 Δ*E*_S_ = *γ*_pv_Δ*A*_pv_ + *γ*_sp_Δ*A*_sp_ + *γ*_sv_Δ*A*_sv_

where Δ*A*_pv_, Δ*A*_sp_, and Δ*A*_sv_ refer to the variations of area of the pollutant-vapor, the solid-pollutant, and the solid–vapor interfaces, respectively. In the process of PM particles deforming and spreading over, the area of the solid–vapor interface reduced is equal to that of solid-pollutant interface increased. Then the released surface energy is

4 Δ*E*_S_ = (*γ*_sv_ − *γ*_sp_)Δ*A*_sv_ + *γ*_pv_Δ*A*_pv_

During the spreading process, the volume of the PM particle remains unchanged, so the above equation can be transformed as

5 Δ*E*_S_ = 2π*R*(10*d* − 2*r*)(*γ*_sv_ − *γ*_sp_) + (4π*r*^2^ + 2π^2^*Rr* − 2π*L*(*R* + *d*))*γ*_pv_

where *R* and *r* refer to the radius of nanofiber and PM particle before deformation and *L* and *d* refer to the length and thickness of the PM particle after spreading over (details of the equation solution procedure are provided in ESI†). A surface plot of the result was generated by the matplotlib module in Python to visually illustrate the impact of *r* and *R* (Fig. 5h). As the *r* increases, the released surface energy will increase, which favors the viscous dissipation of pollutants and gives kinetic energy to overcome the inertial surface tension forces. Thus, the fluffy soots are able to spread over and surround the fiber axisymmetrically and the nanometer-scale channels formed by the SF-based nonofiber were well reserved.

**Fig. 6:**
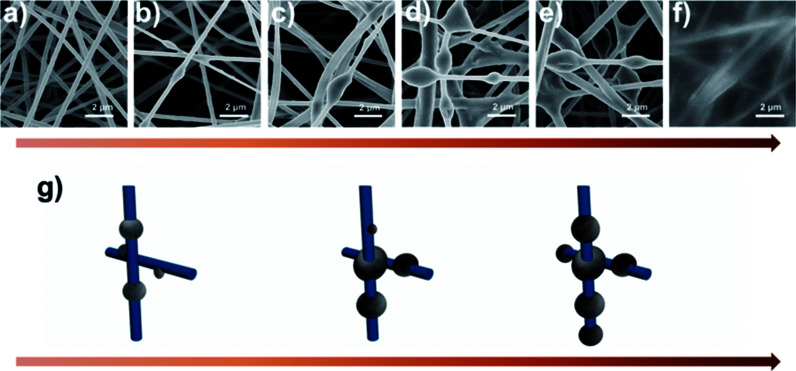
(a–f) SEM images of fluffy soots captured by PAN nanofiber membranes under a continuous flow of smoke at different time sequences. (g) Schematic illustration of the capture, coalescence, and growth of the fluffy soots on PAN nanofiber nets.

For PAN nanofiber air filters, the time sequence of PM capture is shown in panels (a–f) in Fig. 6. As the smoke flowed past the PAN nanofiber, PM particles were first randomly bound around the smooth surface of PAN nonofiber or stuck on one side of the nanofiber only. As the filtration process went on, more PM pollutants were trapped by the nonofiber membrane. Then the small particles aggregated to form larger ones and finally reach to a stable spherical shape, forming beaded fibers and blocking some of the sinuous channel eventually. Fig. 6g depicts a simplified illustration of the capture, coalescence, and growth process of the fluffy soots on PAN nanofiber air filter. When calculating the energy of this system, the gravitational potential energy is neglected as well. During the coalescence process, the surface area of the two small PM particle contracts and the volume stays unchanged. So the variation of interfacial energy in this process can be written as:

6 Δ*E*_S_ = *γ*_pv_Δ*A*_pv_ + *γ*_sp_Δ*A*_sp_ + *γ*_sv_Δ*A*_sv_

where *γ*_pv_, *γ*_sp_, and *γ*_sv_ are the interfacial tension of pollutant-vapor, solid-pollutant, and solid–vapor, respectively. Δ*A*_pv_, Δ*A*_sp_, and Δ*A*_sv_ refer to the variations of area of the pollutant-vapor, the solid-pollutant, and the solid–vapor interfaces. According to the classical Young–Dupre equation^38^ that

7 cos *θ* = (*γ*_sv_ − *γ*_sp_)/*γ*_pv_

then the above equation can be transformed as:

8 Δ*E*_S_ = *γ*_pv_Δ*A*_pv_ − *γ*_pv_ cos *θ*Δ*A*_sp_

with Δ*A*_pv_ < 0, cos *θ* < 0, Δ*A*_sp_ < 0, and the interfacial tension being a positive number. After a simple calculation, we can state that Δ*E*_S_ < 0 always holds. In other words, the fluffy soots can deform and aggregate spontaneously with the release of surface tension as driving force. With the continuous feeding of smoke, more pairs of adjacent fluffy soots coalesced into larger ones to minimize their surface energy. As a result, the air-flow resistance increases because the sinuous channels were blocked by these big filter cake. According to the experimental phenomena and analysis of energy law as we demonstrated above, the excellent filtration performance of SF-based fibrous filtration media is mainly related to the surface tension of silk fibroin and the adhesion between pollutants and SF nanofibers. Silk fibroin is enriched of various functional groups, including hydroxyl (–OH), amide bond (–CO–NH–), phenolic hydroxyl (–C_6_H_4_OH) *etc.*, which are excellent target sites for the coupling of various particles or chemicals. Pollutants could easily deform and spread on the hydrophilic SF nanofibers but they tend to aggregate, contract and finally form ball-shaped conformations on the hydrophobic PAN nanofibers. Compared with the well reserved pathways inside the SF nanofiber membrane, the sinuous channels inside the PAN nanofibers membrane were heavily blocked. In short, the conformation difference of the pore structures between SF membrane and PAN membrane during the filtration process plays a key role in changing the air flow streams and thus affecting the pressure drop.

**Fig. 7:**
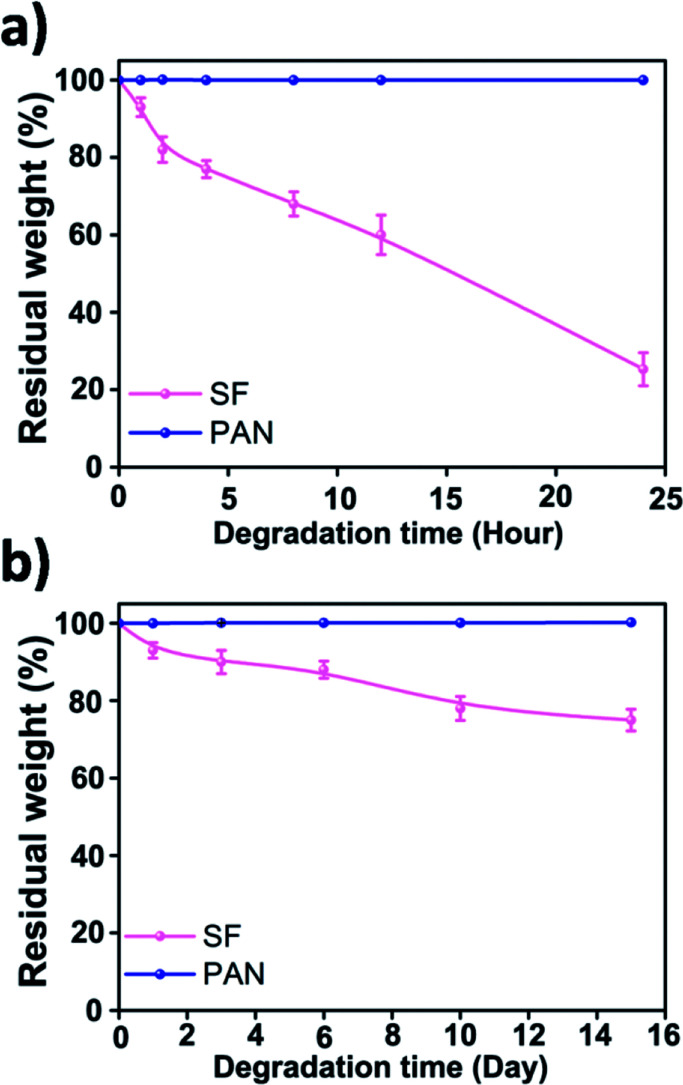
Enzymatic degradation of silk fibroin and PAN nanofiber membranes in PBS solution with protease XIV (a) and in PBS solution only (b).

There are two main waste management strategies, landfilling and incineration. Each of these methods has dangerous side effects. Landfilling is prone to producing toxins, leachate and toxic gases while burning waste emits toxic gases and particulates into the air. When a traditional air filter runs out of its use time, it will be disposed and burned, making the air pollution much worse. Here, we shed a light on the biodegradability of the fibrous filtration media. *In vitro* degradation was carried out by incubating the SF and PAN nanofiber membrane in 50 ml of phosphate-buffered saline (PBS) containing 2.3 U ml^−1^ protease XIV at 37 °C. The results (Fig. 7) indicated that after incubating in protease XIV solution for 24 h, weight loss of the SF nanofiber membrane was about 78% while there was a loss of about 20% in weight after 2 weeks in PBS solution, which can be attributed to the degradation of the residual silk I and/or non-crystalline regions in silk fibroin nanofiber. We should mention that PAN nanofiber membrane incubated in buffered protease XIV solution or PBS only were stable through the whole incubation period. Compared with the synthetic polymer filter, SF air filter can directly degrade into peptides and amino acids, which is non-toxic and can be easily metabolised by microorganisms and macroorganisms in natural environment.^39–41^ This indicates that the biodegradation products of silk fibroin materials do less or no harm to the environment. As shown above, silk fibroins biomaterials are desirable for air filtration because it offers the possibility to be eliminated in the natural environment.

## Conclusions

In summary, eco-friendly membranes based on silk fibroin for high-performance filtration media have been successfully fabricated by a green electrospinning process, which uses water as solvent. This nanofiber air filter showed a superior filtration performance than the state-of-the-art due to its richness in functional groups and good wettability to pollutants. Significantly, a mechanism describing the air flow resistance was proposed based on the principle of energy minimization. Ultimately, the resultant silk fibroin fibrous filtration media are able to improve the filtration efficiency (99.99%) for particle matter with a broad range of size from 0.3 μm to 10 μm while decrease the pressure drop (75 Pa) and present the advantage to be biodegradable. We envision that the green fabrication process and the use of sustainable material would pave the way for the next-generation air filtration system, which exhibited robust filtration performance, excellent biodegradability, and cost-effective.

## Conflicts of interest

There are no conflicts to declare.

## Supplementary Material

RA-008-C7RA12879G-s001
