# Supplementary material for: A silk fibroin based green nano-filter for air filtration
Source: RSC Adv. 2018 Feb 20;8(15):8181–9. doi: 10.1039/c7ra12879g (PMC9078515; doi:10.1039/c7ra12879g)
Supplement: RA-008-C7RA12879G-s001 [file RA-008-C7RA12879G-s001.pdf]

Electronic Supplementary Material (ESI) for RSC Advances.
This journal is © The Royal Society of Chemistry 2018

Supporting Information

Silk Fibroin Based Green Nano-filter for Air Filtration

Xiaochao Gao,[a] *Jing Gou,*[a] *Ling Zhang,**[a] *Shasha Duan,*[a] *Chunzhong Li**[a]

[a] School of Materials Science and Engineering, Key Laboratory for Ultrafine Materials of Ministry of Education, East China University of Science and Technology, 130 Meilong Road, Shanghai 200237, P. R. China

Email: zlingzi@ecust.edu.cn; czli@ecust.edu.cn

Content:

Supplemental Figure Legends: Fig S1-S2;

Supplemental Analysis 1.

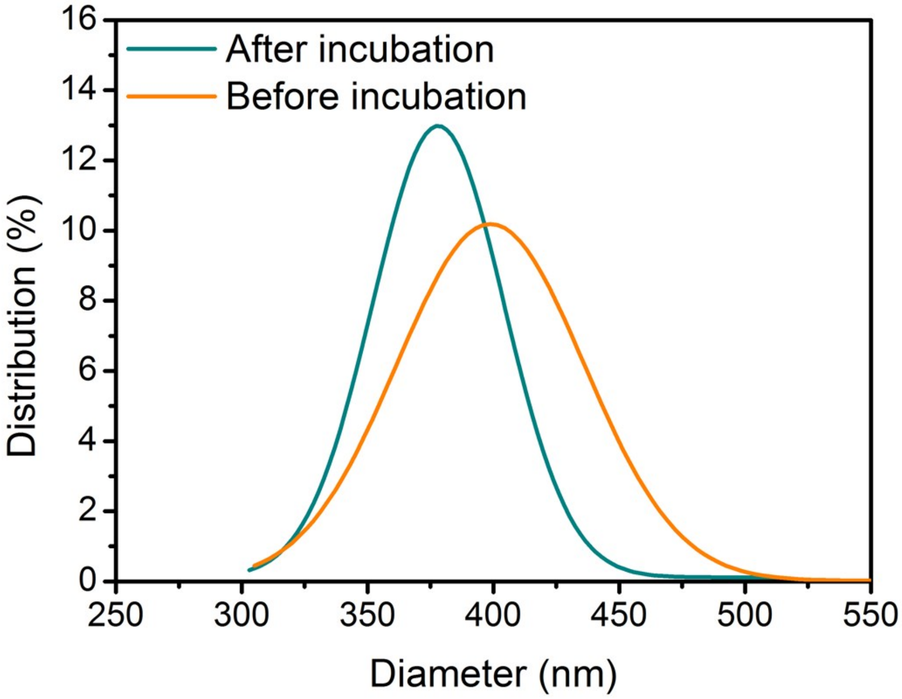

Fig S1. Diameter distribution of SF/PEO blend nanofiber before and after water incubation.

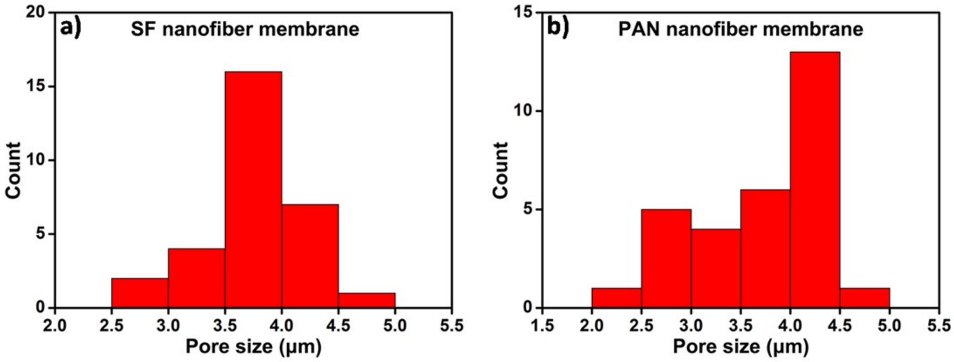

Fig S2. Pore size distribution of (a) SF nanofiber membrane and (b) PAN nanofiber membrane.

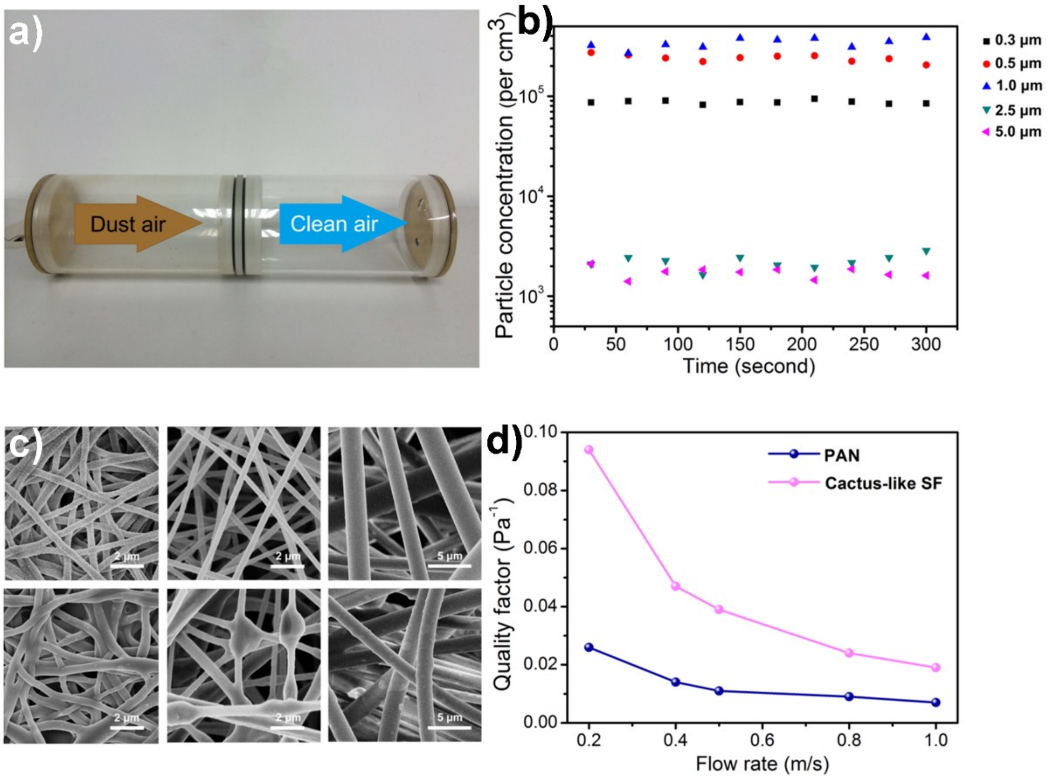

Fig S3. (a) Demonstration of using electrospinning nanofiber filter to shut off PM from the outdoor (right bottle) from entering the indoor (left bottle) environment. (b) Size distribution of PM particles generated by incense burning over time. (c) SEM images of SF, PAN, and commercial transparent filters before and after filtration. (d) Quality factor of SF and PAN nanofiber filters under various air flow velocities.

Supplementary Analysis 1:

As the particles were captured by the nanofiber net, they are able to deform and spread over the nanofiber surface. We assume that the shape of PM pollutants accumulated on the nanofiber net was a solid of revolution. The volumes of PM pollutants can be calculated as:

$$V = \int_{-r}^{r} \pi y^2 dx$$

In addition, the surface area of the PM pollutants is:

$$S = 4\pi r^2 + 2\pi^2 R r$$

After deforming and spreading on the silk fibroin nanofibers, the volume of particles remains unchanged and we assume that the shape after deformation process is a cylinder.

For the filter cake captured by the filtration medium, the general form of the energy is

$$E = \oiint \gamma_{pv} \cdot dA_{pv} + \oiint \gamma_{sp} \cdot dA_{sp} + \oiint \gamma_{sv} \cdot dA_{sv} + \iiint \rho g h \cdot dV$$

where γ_{pv}, γ_{sp}, and γ_{sv} are the interfacial tension of pollutant-vapor, solid-pollutant, and solid-vapor, respectively. A_{pv}, A_{sp}, and A_{sv} refer to the contact area between the pollutant-vapor, the solid-pollutant, and the solid-vapor interfaces. The fourth term represents the gravitational potential energy and once the pollutants are captured by the nanofiber, the variation of gravitational potential energy can be neglected. According to the principle of energy minimization, the surface energy released in the deformation process is

$$\Delta E_S = \gamma_{pv} \cdot \Delta A_{pv} + \gamma_{sp} \cdot \Delta A_{sp} + \gamma_{sv} \cdot \Delta A_{sv}$$

where ΔA_{pv}, ΔA_{sp}, and ΔA_{sv} refer to the variations of area of the pollutant-vapor, the solid-pollutant, and the solid-vapor interfaces, respectively.

In the process of PM particles deforming and sperading over, the area of the solid-vapor interface reduced is equal to that of solid-pollutant interface increased. Then the released surface energy is

$$\Delta E_S = (\gamma_{sv} - \gamma_{sp}) \Delta A_{sv} + \gamma_{pv} \Delta A_{pv}$$

According to the volume and surface area calculated above, the above equation can be transformed as

$$\Delta E_S = 2\pi R\,(10d - 2r)\,(\gamma_{sv} - \gamma_{sp}) + (4\pi r^2 + 2\pi^2 R r - 2\pi L(R + d))\,\gamma_{pv}$$

In order to visually illustrate the impact of r and R, we applied a meshgrid function in Python and generated a surface plot of the equation. As the r increases, the released surface energy will increase, which favors the viscous dissipation of pollutants and gives kinetic energy to overcome the inertial surface tension forces.
